# The Safety of Peripheral Nerve Blocks: The Role of Triple Monitoring in Regional Anaesthesia, a Comprehensive Review

**DOI:** 10.3390/healthcare12070769

**Published:** 2024-04-01

**Authors:** Marek Paśnicki, Andrzej Król, Dariusz Kosson, Marcin Kołacz

**Affiliations:** 1 Department of Anaesthesiology and Intensive Care Education, Medical University of Warsaw, 4 Oczki Str., 02-005 Warsaw, Poland; marek.pasnicki@wum.edu.pl (M.P.); dariusz.kosson@wum.edu.pl (D.K.); 2 Department of Anaesthesia and Chronic Pain Service, St George’s University Hospital, Blackshaw Road Tooting, London SW17 0QT, UK; 3 1st Department of Anaesthesiology and Intensive Care, Medical University of Warsaw, 4 Lindleya Str., 02-005 Warsaw, Poland; marcin.kolacz@wum.edu.pl

**Keywords:** regional anaesthesia, plexus block, peripheral nerve block, pain treatment, interventional pain management, injection pressure, nerve localization

## Abstract

Regional anaesthesia, referred to as regional blocks, is one of the most frequently used methods of anaesthesia for surgery and for pain management. Local anaesthetic drug should be administered as close to the nerve as possible. If administered too far away, this may result in insufficient block. If it is administrated too close, severe nerve damage can occur. Neurostimulation techniques and ultrasound imaging have improved the effectiveness and safety of blockade, but the risk of nerve injury with permanent nerve disfunction has not been eliminated. Intraneural administration of a local anaesthetic damages the nerve mechanically by the needle and the high pressure generated by the drug inside the nerve. In many studies, injection pressure is described as significantly higher for unintended intraneural injections than for perineural ones. In recent years, the concept of combining techniques (neurostimulation + USG imaging + injection pressure monitoring) has emerged as a method increasing safety and efficiency in regional anaesthesia. This study focuses on the contribution of nerve identification methods to improve the safety of peripheral nerve blocks by reducing the risk of neural damage.

## 1. Introduction

Regional anaesthesia, sometimes referred to as regional blocks, is one of the most frequently used methods of anaesthesia both for surgery and for pain management.

Regional anaesthesia as a solo technique or in combination with general anaesthesia has its advantages, providing superior intra- and postoperative pain control and reducing opioid use and the risk of persistent postoperative pain. Efficient cooperation and education of the surgical team is essential in the smooth conduction of the operating list. Patients’ beliefs and anxiety can be addressed by an information package prior to the surgery. Conscious sedation is very helpful in alleviating patient anxiety. Nevertheless, patient refusal remains a primary contraindication.

Blocks are divided into central neuraxial blocks (epidural and subarachnoid anaesthesia) and peripheral nerve blocks. Over the years, improvements in the methods of performing peripheral nerve blocks have been made to ensure that the anaesthesia performed is increasingly effective, to reduce the percentage of failed blocks and to minimize the risk of complications.

On the portal “Anaesthesia Tutorial of the Week” from 2009 Kim Russon et al. lists potential complications of peripheral nerve block (Table 1).

A threat to the patient’s life may occur only in the case of unintentional intravascular administration of a drug causing central nervous system and cardiovascular dysfunction (the so-called Local Anaesthetic Systemic Toxicity (LAST) syndrome). Complications, such as permanent (complete and irreversible) nerve damage, are not life-threatening for the patient, but significantly worsen the patient’s future quality of life [2,3].

A prerequisite for performing effective regional anaesthesia is to administer a local anaesthetic drug into the vicinity of the nerve. The term “in the vicinity” is crucial. This means that the local anaesthetic drug should be administered as close to the nerve as possible. If it is administered too far away, inadequate analgesia or even no analgesia at all may result, while if it is administered too close, or the drug is inadvertently injected into the nerve, it may result in one of the most serious complications that can occur during a procedure of this kind, i.e., permanent nerve damage with accompanying neurological deficits. The following part of the paper introduces the techniques that have made the performance of blocks more effective by enabling the identification of nerves (neurostimulation techniques and ultrasound) and a technique that is now being developed to minimize the risk of nerve damage, i.e., injection pressure monitoring. We focused on the contribution of nerve identification methods to improve the safety of peripheral nerve blocks in terms of damage to neural structures.

## 2. Regional Anaesthesia

Regional anaesthesia involves the reversible blocking of the conduction of pain stimuli in nerve fibres by injecting local anaesthetic drugs into their vicinity.

A nerve is a bundle of parallel nerve fibres, or axons. The individual fibres of the axons are surrounded by myelin and a connective tissue sheath, called the endoneurium. The axons forming the bundles are surrounded by perineurium made up of several layers of concentrically arranged connective tissue cells. Stimulus transmission in nerve fibres is carried out by a wave of depolarization of the cell membrane of the neurons that make up a given nerve. The opening of ion channels and the flow of potassium ions from the cell outward and sodium ions in the opposite direction causes the spread of the nerve impulse [4].

Local anaesthetics block the sodium channels and cause the inhibition of impulse (signal) conduction. The nerve is a delicate structure, and damaging it with a needle or injecting the drug into the nerve itself can cause irreversible damage. The local anaesthetic drug must reach the axon, which means that it must be administered as close as possible, while avoiding potentially dangerous contact with the needle.

The amount of administered regional anaesthesia in surgery varies and depends on the specifics of the procedure. There are specialties where all the procedures are performed under general anaesthesia (neurosurgery and cardiothoracic surgery), but, for example, in the case of orthopaedic procedures, especially of the extremities, the number of cases when regional anaesthesia is administered by far exceeds that of using general anaesthesia. The percentage of cases when regional anaesthesia is performed ranks particularly high in centres with extensive experience in this field and, thus, is characterized by high safety and high efficiency [5].

Thanks to regional anaesthesia techniques, general anaesthesia can be avoided in patients for whom the latter is associated with high risk.

### 2.1. Selected Advantages and Disadvantages of Peripheral Nerve Blocks

It has been proven that peripheral nerve blocks lower the prevalence of postoperative pain and reduce the perioperative needs for opioids and the adverse effects of general anaesthesia, including nausea and vomiting [6].

Postoperative pain management is facilitated due to the long duration of the blocks and the slow emergence from anaesthesia [7]. Continuous nerve blocks with implanted temporary catheters are also an effective way to continue peripheral block in the postoperative period [8].

On the other hand, regardless of other potential causes, peripheral nerve blocks can be responsible for neurological complications. Studies estimate that even up to 14% of neurological complications occur after brachial plexus block [9].

Nerve damage has a rather complex pathophysiology. Seddon and Sunderland’s (Table 2 and Table 3) classification analyses the degree of damage and the chance of spontaneous return of function.

The mildest form of damage is neurapraxia. Nerve dysfunction is caused by the swelling of the axon, disorganization of neurofilaments, and segmental demyelination. After 2 to 12 weeks, there should be a full return of conduction. Axonotmesis is an interruption of the continuity of the axons, and depending on whether the epineurium and perineurium have also been damaged, the prognosis for the return of full function is worse. The most severe complication is complete transection of the nerve along with all the connective tissue elements (neurotmesis). Spontaneous return of function without surgical intervention is impossible [10,11].

Transient neurologic symptoms (TNSs) are characteristic after central blocks especially with lidocaine [12,13,14]. Unfortunately, transient neurological disorders (TNDs) are also relatively common in the first days after peripheral nerve blocks and their frequency of occurrence from approximately 0.3% (femoral nerve block) to 2.8% (brachial plexus block) [15].

The cause of TNDs may be reversable neuropraxia (grade 1 in Sunderland’s classification) as a result of transient compression of the nerve.

### 2.2. Permanent Nerve Injury

Mechanical nerve injury after peripheral nerve blocks can be caused by the needle (cut, tear), compression from outside (high volume of anaesthetic solution, hematoma of the adjected tissues), and intraneural injection [16].

It should be noted that the mechanisms of peripheral nerve injury can be divided into four groups: chemical, mechanical, vascular, and inflammatory. These may be associated not only with anaesthetic, but also surgical factors and patients’ predisposition [17].

Intraneural administration of a local anaesthetic damages the nerve both mechanically (severance of the fibres) and through the high pressure generated by the drug administered into the closed space surrounded by the epineurium. If, at the same time, damage is done to the blood vessels in the nerve (vasa nervorum) and an intraneural hematoma is formed, the risk of permanent damage increases significantly [18].

### 2.3. Prevalence of Permanent Nerve Injury

Data from the literature are disparate and largely depend on the definition and criteria adopted. It has been agreed that a neurological deficit lasting more than a year is considered permanent damage. Deficits that disappear after a few days are much more common than permanent paralysis. A meta-analysis that appeared in 2007 reviewed 32 studies published between 1995 and 2005 aiming to assess the risk of neurological complications after central and peripheral nerve blocks. The incidence of neuropathy after subarachnoid anaesthesia is 3.78 per 10,000 cases and, after epidural anaesthesia, 2.19 per 10,000. For peripheral nerves and plexus anaesthesia, the incidence of neuropathy after brachial plexus block between the oblique muscles was 2.84 per 100 cases, while from axillary access, it was 1.48 per 100, whereas for femoral nerve block, 0.34 per 100. It should be assumed that peripheral nerve anaesthesia is relatively safe and life-threatening complications do not occur; however, neurological complications occur statistically more often than in the case of central blocks [15].

The most commonly reported incidence of persistent (over a year) of neurological injuries associated with regional anaesthesia is 2–4 per 10,000 blocks [1,18,19,20,21,22].

The classical description of RA blocks relied on the anatomical knowledge of superficial landmarks and deeper lying structures, as per anatomy textbooks. Precise “technique recipes” were published on how to approach specific neural targets. Examples are the approach to the brachial plexus described by Hirschel or Winni [23,24,25].

The success of a blockade depended largely on the experience and skill of the person who performed the procedure and his/her knowledge of anatomy and spatial imagination.

The injection site was determined on the basis of locating anatomical points, and the correct position of the needle was confirmed by the painful and unpleasant paresthesias felt by the patient and caused by direct needle prick of the nerve. Paraesthesia was even considered as an indicator of block effectiveness (“no paraesthesia no anaesthesia” or “no paraesthesia no dysaesthesia, but of the failed anaesthesia”) [26].

When using such a method, the risk of permanent nerve damage was about 3% [27].

Additionally, the effectiveness of regional blocks relying on landmarks and paraesthesia was also low, even when a large volume of local anaesthetic (LA) was used [28].

Even the best regional anaesthetists were not able to appreciate individual patients’ anatomical variations.

For effective and safe conduction of regional anaesthesia, the following requirements are to be met:

1. Careful planning of needle site entry to avoid vital structures such as vessels, pleura, viscera, or other nerve structures not being a target of intervention.
2. Precise administration of LA close to the nerve structure, but outside the epineurium to reduce the total volume of LA and the risk of LAST.
3. Avoidance of direct nerve injury by the needle and intraneural injection of LA.

Attempts are made to meet these requirements by using techniques that facilitate nerves and other structures’ localization without damaging them.

### 2.4. Nerve Localization Techniques

Similar to other methods in this field, regional anaesthesia has been evolving into using more reliable, safer methods relying on new devices and techniques.

### 2.5. Electrostimulation of Nerves

The first step was the introduction of a nerve stimulator and needles designed specifically for this technique. Reports on the use of electrostimulation and highlighting its advantages appear as early as the 1970s. The advantages of the new technique, such as the reduced risk of nerve damage and the lower dose of local anaesthetic drug that needed to be administered, especially when compared to the large-volume drug delivery techniques used previously, was emphasised by Chapman in 1972 [7,29].

A nerve stimulator is a device that generates electrical pulses of adjustable intensity and frequency. A current with a fixed pulse duration (usually 0.1 ms) and a fixed frequency (1 or 2 Hz) is most frequently used. Only the intensity of the pulse changes during the execution of the blockage. A specially designed needle, which is insulated, except for the tip, which works with the stimulator, makes it easier to find the relevant nerve structures by observing the motor response from the muscles. Changes in current intensity as the nerve is approached and the observation of the motor response to the electrical impulses makes it possible to guide the needle very precisely, so that the smallest stimulation current that elicits a response is within 0.2–0.5 mA [30].

It is important to remember a number of limitations of this method. The electric current always follows the path of least resistance, and the electrical impulse may follow a path that is not at all the shortest [31]. This can give the effect of “false positive and false negative stimulation” and result in an inadequate analgesic effect of the blockade.

It has also been reported that a lack of motor response to stimulation does not rule out intraneural needle placement [32].

Ultrasonography turned out to be a milestone in achieving the quality and safety of regional anaesthesia.

### 2.6. Ultrasonography

In recent years, ultrasound imaging has become widespread in many medical specialties, including anaesthesiology. It has improved both vascular cannulation and the performance of regional anaesthesia. Direct observation of anatomical structures and the possibility to visualize the exact position of the needle have increased the efficiency of the blocks performed, reduced the risk of nerve damage or improper drug administration (including the extremely dangerous intravascular administration), and made it possible to reduce the volume of local anaesthetics administered. The so-called “volumetric technique” is being abandoned, i.e., the method of administering a large volume of drug to increase the effectiveness of the blockade even in cases of insufficiently precise injection.

**Figure 1:**
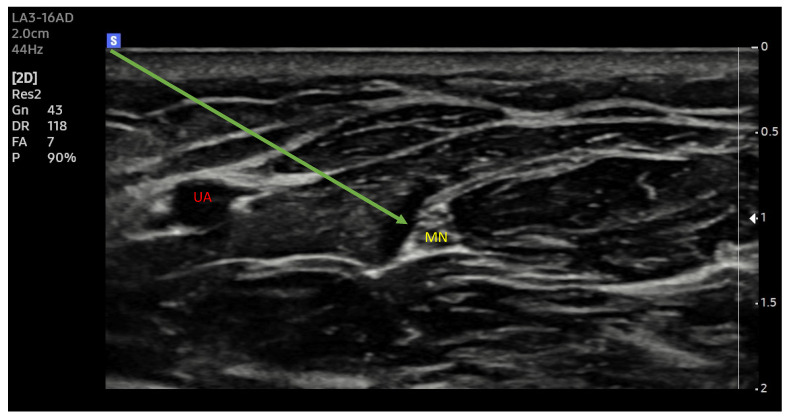
Median nerve. Ultrasound transverse view. MN—median nerve, UA—ulnar artery; Arrow —needle trajectory; Arrowhead—extraneuronal needle placement.

**Figure 2:**
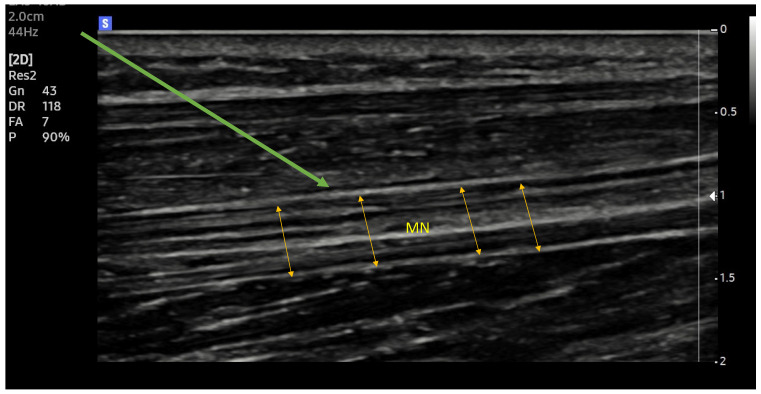
Median nerve. Ultrasound longitudinal (long axes) view. MN (yellow arrows)—median nerve; Green arrow—needle trajectory; Arrowhead—extraneuronal needle placement.

New ultrasound machines with digital image processing and customized image parameter settings make it easier to identify the selected structures (Figure 1 and Figure 2). Increasing the precision of delivery into the immediate vicinity of nerves increases the effectiveness of anaesthesia and makes it possible to reduce the dose of local anaesthetic drugs [33].

Ultrasound nerve identification compared with nerve stimulation techniques reduced the number of needle passes needed to perform the block (from 3 to 1); however, significant differences in block failures were not observed. Both procedures were effective, and no patients required conversion to general anaesthesia [34]. The incidence of unintended intraneural administrations during ultrasound-guided brachial plexus blocks performed by experienced physicians is up to 17% [35,36].

Undoubtedly, it is still necessary to look for methods that will increase the safety of performing blocks and reduce the risk of permanent nerve damage [37].

### 2.7. Injection Pressure Monitoring

Nerve injury may be indicated by the patient’s report of pain at the time of injection or by very unpleasant paraesthesia. Under no circumstances should the drug be administered when clear resistance is felt on the syringe plunger. The term “clear resistance” is imprecise. It should be clarified, and acceptable pressure values should be introduced during injection.

A commonly used kit for blocks consists of a syringe, a drain connecting the needle and syringe, and a needle. The resistance to the flow of the fluid generated by the needle and the drain is proportional to their length and inversely proportional to their diameter. Also, the pressure generated by the syringe depends on its diameter and, thus, the surface area of its plunger. It is a well-known phenomenon that, by using a smaller syringe, it is easier to overcome the resistance posed by a long catheter with a small diameter and the drug is administered using less force. Thus, there are quite a few variables, so it is difficult to determine precisely what pressures are in fact generated in such a system.

Considerable resistance to the administration of the drug may indicate that the injection is given to the nerve and not in its vicinity. Therefore, it seems reasonable that, for the safety of the patient and to avoid permanent damage to the nerve, this pressure should be monitored. It is necessary to determine the permissible pressure values at which the injection can be performed. If such values are exceeded, the drug may have been injected into the nerve.

Experiments on animals and on preparations from human cadavers have proven invaluable. The latter are properly preserved either chemically or with the use of a low temperature to reflect the anatomy and structure of living human tissues as closely as possible. This makes it possible to safely simulate under laboratory conditions such situations as, for example, unintentional nerve damage with a needle.

A study published in 2018 described testing an injection-pressure-monitoring system. The system consisted of a miniature pressure sensor integrated into the needle, which in turn, was connected to the control (monitoring) unit by a fibre optic cable. The system’s recurrent pressures were monitored and interpreted when performing a sciatic nerve block on a fresh cadaver. The trials included 24 ultrasound-guided injections: 12 perineural and 12 intraneural ones. It was shown that the peak injection pressure was significantly lower for perineural injections (the mean was 14 kPa), as compared to intraneural injections (the mean was 131 kPa). This study demonstrates that the proposed system is a good solution to the problem of monitoring the injection pressure at the end of the needle, making it possible to distinguish between perineural and intraneural injections [38].

It also defines the concept of pressure needed to initiate an injection, i.e., to overcome the resistance of the tissues surrounding the needle tip. In the subsequent stage of injection, the pressure will depend on various factors, primarily the speed of injection, but also the gauge of the needle [39]. Monitoring the pressure in the local anaesthetic drug delivery system makes it possible not only to detect intraneural administration, but also to warn against excessively close needle–nerve contact (NNC). In a cadaveric study published by A. Krol and colleagues, the pressures for the median, radial, and ulnar nerves were analysed. Experimentally, 60 injections were performed (30 intraneural and 30 perineural). The results of this study demonstrated differences between intraneural and perineural injection pressure. All perineural injections produced pressures < 12 psi [40]. The same authors have also demonstrated statistically significant differences between intraneural and perineural injection pressures [41].

A study published in 2014 describes a procedure for monitoring injection pressure and attempted to find a correlation between NNC and needle–nerve contact that is too close. The study was conducted on patients undergoing shoulder surgery for which brachial plexus block was performed. It was shown that the pressure that was needed to initiate the injection (OIP) was less than 15 psi, while direct contact of the needle with the nerve generated a pressure increase of more than 15 psi [42].

In most studies, a pressure of 15 psi was considered the limiting pressure above which injection should not be performed. Can this value be taken as a universal value, common to all nerves? It has been shown in a cadaveric study that pressures at intraneural administration are different for different nerves [40]. It also must be kept in mind that there are variations in the structure of different nerves, primarily differences in the thickness of the entire nerve and of the connective tissue sheath. Viewing a histological preparation, one can notice the different ratio that the nerve bundles occupy in relation to the connective tissue. Thus, anatomical differences may result in different limiting pressures for different areas of the same nerve structure. The highest interneuronal pressure (INIP) was observed for brachial plexus trunks (31.2 ± 6.0 psi). A significantly lower INIP was generated for supraclavicular access (24 ± 15.0 psi) and subclavian access, i.e., 23.4 ± 9.5 psi. The lowest pressures were observed in the tibial nerve, with intraneural administration generating an INIP of 17 ± 7.3 psi [40].

These data, as well as the data obtained by the cadaveric study may be a prelude to the creation of a specific “injection pressure map” and the consideration of block-specific characteristics [40].

Injection pressure monitoring may be useful for the identification not only of nerves, but also the interfascial plane. It has been shown that the characteristic three-peak pattern of pressure was observed during injection. Pressure increases when the needle tip makes contact with the muscle fasciae [43].

Regardless of the evidence that intraneural injection consistently creates a higher pressure than perineural injections, this finding may be limited by the false positive results related to other mechanical obstructions such as bone, tendon, as well as the rate of infusion. An infusion rate above 0.3 mL/s regardless of the needle gauge and length may increase the pressure above 15 psi even during free flow.

**Figure 3:**
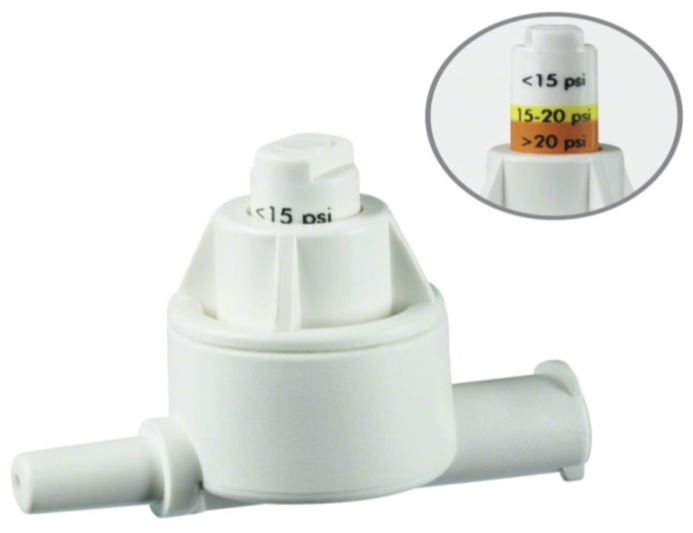
The B-Smart injection pressure monitor device. Reproduced with permission Aesculap Chifa sp. z o.o.-a B.Braun company (Warsaw, Poland).

**Figure 4:**
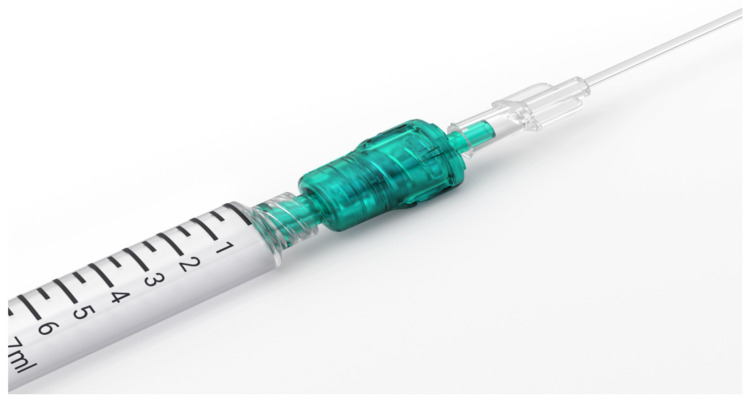
The NerveGuard automatic injection pressure limiter. Reproduced with permission from PAJUNK® GmbH Medizintechnologie (Geisingen, Germany).

**Figure 5:**
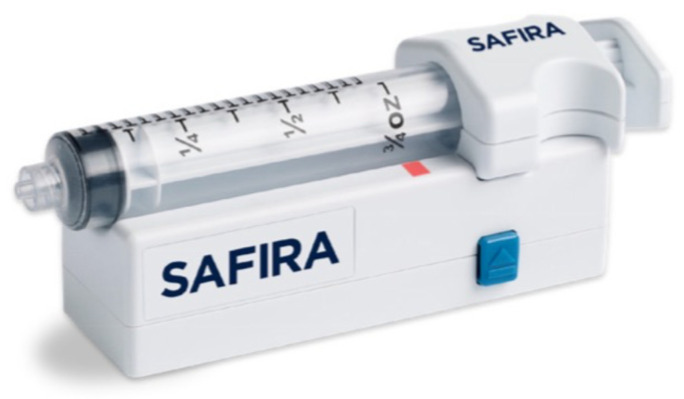
The SAFIRA® device. Reproduced with permission from Vygon Polska (Warsaw, Poland).

Until recently, there were no commercial devices available to monitor injection pressure. Currently, there are at least three devices on the market. The B Braun injection pressure monitor (Melsungen, Germany) is placed between the needle and syringe, popping out when the pressure reaches 15 psi (yellow) or 20 psi (red). One is still able to overcome the pressure and inject (Figure 3). The pressure guard from Pajunk prevents injection (Geisingen, Germany) higher than 15 psi (Figure 4). Both devices are portable and affordable. The third one, Safe Administration of Regional Anesthesia (SAFIRA), has been also validated, but is more complex in application (Figure 5) [44].

### 2.8. Triple Monitoring

There was an idea that, in addition to monitoring pressure during injection, it would be good practice to combine the ultrasound technique with the older nerve stimulation technique. Combining the advantages of both would improve protection against the risk of nerve damage. Ultrasound makes it possible to observe anatomical structures and guiding the needle in a direct way, while nerve stimulation connected to the system (set at 0.2–0.3 mA) would signal if contact between the nerve and the needle was too close, and pressure monitoring during injection would signal the danger of injecting local anaesthetic drugs into the nerve or its sheath [45].

The combination of two modalities: ultrasound and nerve stimulator, so-called dual-guidance, has been advocated by experts in the field [46,47].

It has been reported that ultrasound nerve identification supported by nerve stimulation localizes the target in more than 98% of cases, compared with 90.1% and 81.6% when stimulation and ultrasound techniques were used separately. Also, side effects including intraneuronal needle placement were much less frequent when dual-guidance was used (0.5% vs. 2.5% vs. 4%, respectively) [48].

A paper describing the concept of the combination of these three monitoring techniques titled “Triple monitoring”, i.e., ultrasound + nerve stimulator + pressure monitoring, appeared in 2021 in the *Journal of Clinical Medicine*. It presented the experience of performing brachial plexus block for shoulder arthroscopy surgery. The study group consisted of 60 patients. In this study, brachial plexus block with triple monitoring was performed in 60 patients who underwent arthroscopic shoulder surgery. Observation with ultrasound enabled precise placement of the needle tip between the brachial plexus roots. Using a nerve stimulator set to a pulse intensity of 0.5 mA allowed the exclusion of direct contact between the needle and the nerve root. The final, third stage of control was the monitoring of pressure during the injection attempt. The injection was performed only if the pressure was less than 15 psi. Patients were injected with 10 mL of 0.75% ropivacaine, and all injection procedures were performed by the same anaesthesiologist.

It was shown that dangerously close contact between the needle and the nerve was detected by means of a nerve stimulator in 18 patients (30%), despite the fact that ultrasound visualization did not signal such danger. The injection pressure sensor detected a dangerous increase in pressure in another group of 15 patients, despite the absence of the features of electrical stimulation of the nerve.

During follow-up in the postoperative period (24 h, 7 days, and 1 month), none of the anesthetized patients showed neurological disorders. The conclusion was that triple monitoring does reduce the risk of nerve damage during the block. Ultrasound observation alone may not be sufficiently accurate or reliable, while in some situations, neurostimulation may not signal unintentional nerve puncture [49].

## 3. Conclusions

The combination of all three techniques (the so-called triple monitoring) could improve safety and reduce complications.

The ultrasound enables the real-time visualization of the target nerve, needle, blood vessels, viscera, or other nerve structures not being a target of intervention and anaesthetic solution spread. In contemporary practice, “scout scanning” is often performed before preparing the sterile field for block. The peripheral nerve stimulator helps to confirm the target, but motor response at 0.2 mA warns about the needle being too close or even intraneural. High injection pressure (>15 psi) may suggest an intraneural needle position, but also may indicate a too rapid injection and undesired spread, e.g., neuraxially. Chronic pain blocks of selective spinal nerves and epidural injections in the presence of foraminal and spinal stenosis deserve special attention with pressure monitoring. To date, we have found no reports of nerve injury while using injection pressure monitoring during peripheral nerve block.

Certain anatomical conditions such as a high BMI and postoperative and posttraumatic changes can make particular blocks exceptionality challenging. This has been already highlighted by Krol and De Andres, suggesting a nerve stimulator and injection pressure as standard monitoring along with ultrasound [50].

## Figures and Tables

**Table 1:** Potential complications with any peripheral nerve block. Adapted from: [1] (https://resources.wfsahq.org/atotw/peripheral-nerve-blocks-getting-started/, accessed on 5 March 2024).

Potential Complications with any Peripheral Nerve Block
Block failure
Intravascular injection
Local Anaesthetic Toxicity
Nerve damage: temporary or permanent
Injury secondary to numbness or weakness
Infection

**Table 2:** Seddon’s classification of nerve injury.

Seddon’s Classification	
neurapraxia	Compression of the nerve without breaking its continuity. Nerve conduction is impaired. Symptoms disappear after a few to a few dozen days.
axonotmesis	The nerve is intact, but the axons are damaged. Complete loss of the nerve function occurs. Nerves can regenerate, but this can take up to several dozen months.
neurotmesis	Transection of the nerve causes its paralysis. No rapid regeneration is possible. Surgical intervention is necessary.

**Table 3:** Sunderland’s classification of nerve injury.

Sunderland’s Classification	
grade I	Lack of conduction due to nerve compression Corresponds to Seddon’s neurapraxia
grade II	Breaking of the axon without nerve damage Corresponds to Seddon’s axonotmesis
grade III	Damage of the endoneurium, without changes in the epi- and perineurium Return of function depends on endoneuronal fibrosis
grade IV	Damage to all sheaths apart from the epineurium Enlargement of the nerve may occur
grade V	Complete severance or disruption of the nerve Corresponds to Seddon’s neurotmesis

## Data Availability

The data that support this study are available within the reference or available from the authors upon request.
